# Effects of acute phase intensive electrical muscle stimulation in COVID-19 patients requiring invasive mechanical ventilation: an observational case-control study

**DOI:** 10.1038/s41598-024-55969-8

**Published:** 2024-03-04

**Authors:** Yohei Tsuchikawa, Shinya Tanaka, Daisuke Kasugai, Riko Nakagawa, Miho Shimizu, Takayuki Inoue, Motoki Nagaya, Takafumi Nasu, Norihito Omote, Michiko Higashi, Takanori Yamamoto, Naruhiro Jingushi, Atsushi Numaguchi, Yoshihiro Nishida

**Affiliations:** 1 Department of Rehabilitation, Nagoya University Hospital, Nagoya, Japan; 2 Department of Emergency and Critical Care Medicine, Nagoya University Graduate School of Medicine, Nagoya, Japan; 3 Department of Rehabilitation, Mie University Hospital, Tsu, Japan; 4 Department of Rehabilitation, Juko Osu Hospital, Nagoya, Japan; 5 Department of Respiratory Medicine, Nagoya University Graduate School of Medicine, Nagoya, Japan; 6 Department of Orthopaedic Surgery, Nagoya University Graduate School of Medicine, 65 Tsurumai-cho, Showa-ku, Nagoya, 466-8560 Japan

**Keywords:** Neuromuscular electrical stimulation, Physical performance, Intensive care unit acquired weakness, SARS-CoV-2, Ventilator, Infectious diseases, Respiratory tract diseases, Rehabilitation

## Abstract

We investigated the effects of acute-phase intensive electrical muscle stimulation (EMS) on physical function in COVID-19 patients with respiratory failure requiring invasive mechanical ventilation (IMV) in the intensive care unit (ICU). Consecutive COVID-19 patients requiring IMV admitted to a university hospital ICU between January and April 2022 (EMS therapy group) or between March and September 2021 (age-matched historical control group) were included in this retrospective observational case–control study. EMS was applied to both upper and lower limb muscles for up to 2 weeks in the EMS therapy group. The study population consisted of 16 patients undergoing EMS therapy and 16 age-matched historical controls (median age, 71 years; 81.2% male). The mean period until initiation of EMS therapy after ICU admission was 3.2 ± 1.4 days. The EMS therapy group completed a mean of 6.2 ± 3.7 EMS sessions, and no adverse events occurred. There were no significant differences between the two groups in Medical Research Council sum score (51 vs. 53 points, respectively; P = 0.439) or ICU mobility scale at ICU discharge. Addition of upper and lower limb muscle EMS therapy to an early rehabilitation program did not result in improved physical function at ICU discharge in severe COVID-19 patients.

## Introduction

The capacities of healthcare systems around the world have been stressed by the novel coronavirus disease 2019 (COVID-19) pandemic, which is caused by infection with severe acute respiratory syndrome coronavirus 2 (SARS-CoV-2). Severe COVID-19 requiring invasive mechanical ventilation (IMV) has an estimated in-hospital mortality rate of approximately 45%^1^, with survivors often requiring prolonged IMV support in the intensive care unit (ICU)^2^. Such patients requiring prolonged IMV have prolonged ICU stays and require deep sedation, neuromuscular blockade, and/or placement in the prone position, which are significant risk factors for ICU-acquired weakness^3^, and have high rates of development of impairments in physical function, limited mobility, mental health, and quality of life after discharge from the ICU or from hospital^4–7^.

Early exercise with the active involvement of a physiotherapist is recommended after ICU discharge among patients with COVID-19^8^. Early mobilization and exercise appear to be essential for treatment of severe COVID-19, and recent studies demonstrated the safety and efficacy of early rehabilitation therapy in patients with severe COVID-19 treated in the ICU^9^. However, active early rehabilitation often cannot be performed due to limited medical resources, especially lack of personal protective equipment and personnel required for patients with obesity, severe physical dysfunction, and/or following IMV in the ICU^4,10,11^. Novel interventions are therefore required to prevent early injury and enhance functional recovery of patients with severe COVID-19 requiring treatment in the ICU. A meta-analysis of randomized controlled trials (RCTs) reported that the use of electrical muscle stimulation (EMS)—a method for safely inducing muscle contraction without requiring volitional effort and that does not evoke dyspnea—can reduce the incidence of ICU-acquired weakness in critically ill patients^12–15^. Therefore, EMS is expected to be effective and an adjunctive therapy or a bridge to rehabilitation in patients with COVID-19^16,17^, but its benefits are not clear. The present study was performed to determine whether intensive EMS add-on therapy could improve the muscle strength in patients with COVID-19 requiring IMV in the ICU compared with early rehabilitation alone.

## Methods

### Study cohorts

This single-center, retrospective observational, case–control study was performed in patients ≥ 18 years old admitted to the ICU of Nagoya University Hospital due to COVID-19 with respiratory failure requiring IMV between January and April 2022 (EMS therapy group) and age-matched controls admitted between March and September 2021 (historical control group) with length of stay > 24 h in the ICU. Patients who died in the ICU, who were not intubated, and who did not receive rehabilitation therapy in the ICU were excluded.

In all patients, COVID-19 diagnosis was confirmed by real-time polymerase chain reaction (PCR) for SARS-CoV-2 from any specimen. Our clinical setting and management of COVID-19 were reported previously^5,18^. Management of COVID-19 requiring IMV in the ICU was based on the “ABCDEF (Assess & manage pain, Both spontaneous awakening trials and spontaneous breathing trials, Choice of sedation and analgesia, Delirium assessment & management, Early mobilization and exercise, and Family engagement)” bundle^19^. Patients requiring < 4 L of O_2_ were transferred to the general COVID-19 ward. Rehabilitation therapy was performed by a multidisciplinary critical care team. The first stage of rehabilitation performed in patients with Richmond Agitation Sedation Scale (RASS) score ≤  − 2 consisted of positioning or range of motion exercises. In patients whose condition stabilized, rehabilitation proceeded to the second stage consisting of sitting on the edge of the bed, standing, transferring to a chair, and active muscle training until discharge from the ICU.

### Electrical muscle stimulation

EMS therapy was incorporated into the rehabilitation program in all patients in the EMS therapy group once they had progressed beyond the initial very acute phase after discontinuing neuromuscular blockade. Patients with skin lesions, cardiac pacemakers, infection or trauma of the extremities, those who were unable to walk before hospital admission, and those who could not speak Japanese were excluded from the EMS therapy group. EMS was applied to the bilateral upper and lower limb muscles (biceps brachii, quadriceps femoris, and gastrocnemius muscles: middle of the upper arm and approximately 2 cm above the cubital fossa for biceps brachii, approximately 5 cm below the inguinal fold and 3 cm above the upper patella border for the quadriceps femoris, and approximately 3 cm below the popliteal fossa and immediately above the proximal end of the Achilles tendon for the gastrocnemius muscles) with a stimulator (Solius; Minato Medical Science, Osaka, Japan) using self-adhesive surface electrodes (40 × 80 mm). The EMS intervention included as part of the standard rehabilitation therapy for patients with respiratory or circulatory failure and postoperative patients in the ICU in our institution was reported previously^20–22^. We applied EMS with a variable-frequency train that began with high-frequency bursts (200 Hz), followed by low-frequency stimulation (20 Hz), and EMS was applied as a symmetrical biphasic square wave with 0.4-s pulses of direct current followed by a 0.6-s pause. Pulse groups consisting of 10 impulse trains were delivered to unilateral muscle groups at 10-s intervals during the session, and the output current was adjusted to ensure visible muscle contraction. EMS was applied by trained physiotherapists for 30 min per day, 6 days per week, for up to 2 weeks until the discharge from the ICU. We set the discontinuation criteria during the EMS session as follows: (1) change in systolic blood pressure >  ± 20 mmHg; (2) increase in heart rate >  + 20 beats/min; (3) development of sustained ventricular arrhythmia, atrial fibrillation, and paroxysmal supraventricular tachycardia; (4) decrease in blood oxygen saturation > − 4%.

### Data collection

The Coronavirus Clinical Characterisation Consortium Mortality Score was calculated for each patient on admission to the ICU^23^. The worst Acute Physiology and Chronic Health Evaluation II (APACHE II) and Sequential Organ Failure Assessment (SOFA) scores, both of which were also calculated within 24 h after ICU admission, were used in the analyses. The clinical frailty scale was used to assess the degree of frailty prior to ICU admission, with scores ranging from 1 (very fit) to 9 (terminally ill)^24^.

### Physical function and clinical outcomes

Physical function was evaluated in each patient at the time of discharge from the ICU. Muscle strength was determined based on the Medical Research Council (MRC) sum score, which assesses the strength of each muscle group in the upper and lower limbs with scores for each muscle group ranging from 0 to 5 and higher scores indicating greater muscle strength (total score range: 0 = worst to 60 = best, minimal clinically important difference 4 points)^3,25^; MRC sum score < 48 points was taken as the definition of muscle weakness^26^. Handgrip strength was also measured to assess muscle strength with the patient performing two maximal isometric voluntary contractions of each hand for 3 s with the elbow joint fixed at 90° flexion in the supine position using a Jamar dynamometer set to the second handle position (DHD-1 Digital Hand Dynamometer; Saehan Corporation, Seoul, South Korea). The greatest strength expressed as an absolute value (kg) was used in the analyses. The grip and release test and foot tapping test, involving measurement of the number of times the patient could flex and stretch the fingers of each hand in 10 s and tap the sole of each foot in 10 s while keeping the heel in contact with the floor and with the knees at 90° flexion, were performed with the patient in the supine position to evaluate upper and lower peripheral extremity motor function, respectively^27,28^. The analyses were performed using the highest scores obtained for both grip and release test and foot tapping test.

Clinical outcomes, including length of stay in the ICU, unplanned readmission to the ICU, and the location of hospital discharge (i.e., home or to another department/institution/ward/facility), were included in the analysis. At ICU discharge, we calculated the ICU mobility scale score for each patient determined on an 11-point ordinal scale ranging from 0 (lying/passive exercises in bed) to 10 (independent ambulation). The time taken to first mobilization (defined as ICU mobility scale score ≥ 3, i.e., sitting on the edge of the bed or higher) was assessed^29^.

### Statistical analysis

Continuous variables are expressed as the median and interquartile range (IQR), and categorical variables are expressed as numbers and percentages. Differences between groups were evaluated by the Mann–Whitney U test for continuous variables and Fisher’s exact test for dichotomous variables. The primary outcome was MRC sum score at ICU discharge.

Statistical analyses were performed using SPSS version 23.0 (IBM Corp., Armonk, NY) and R version 3.2.1 (R Foundation for Statistical Computing, Vienna, Austria). In all analyses, a two-tailed P < 0.05 was taken to indicate statistical significance.

### Ethics approval and consent to participate

This study was approved by the Institutional Review Board of Nagoya University Hospital, and was performed in accordance with the tenets of the Declaration of Helsinki and the Japanese Ethical Guidelines for Medical and Health Research Involving Human Subjects. Informed patient consent was obtained, and the patients agreed to reveal their facial photos for academic purposes. All participants were informed that they were free to opt out of participation in the study at any time.

## Results

**Figure 1:**
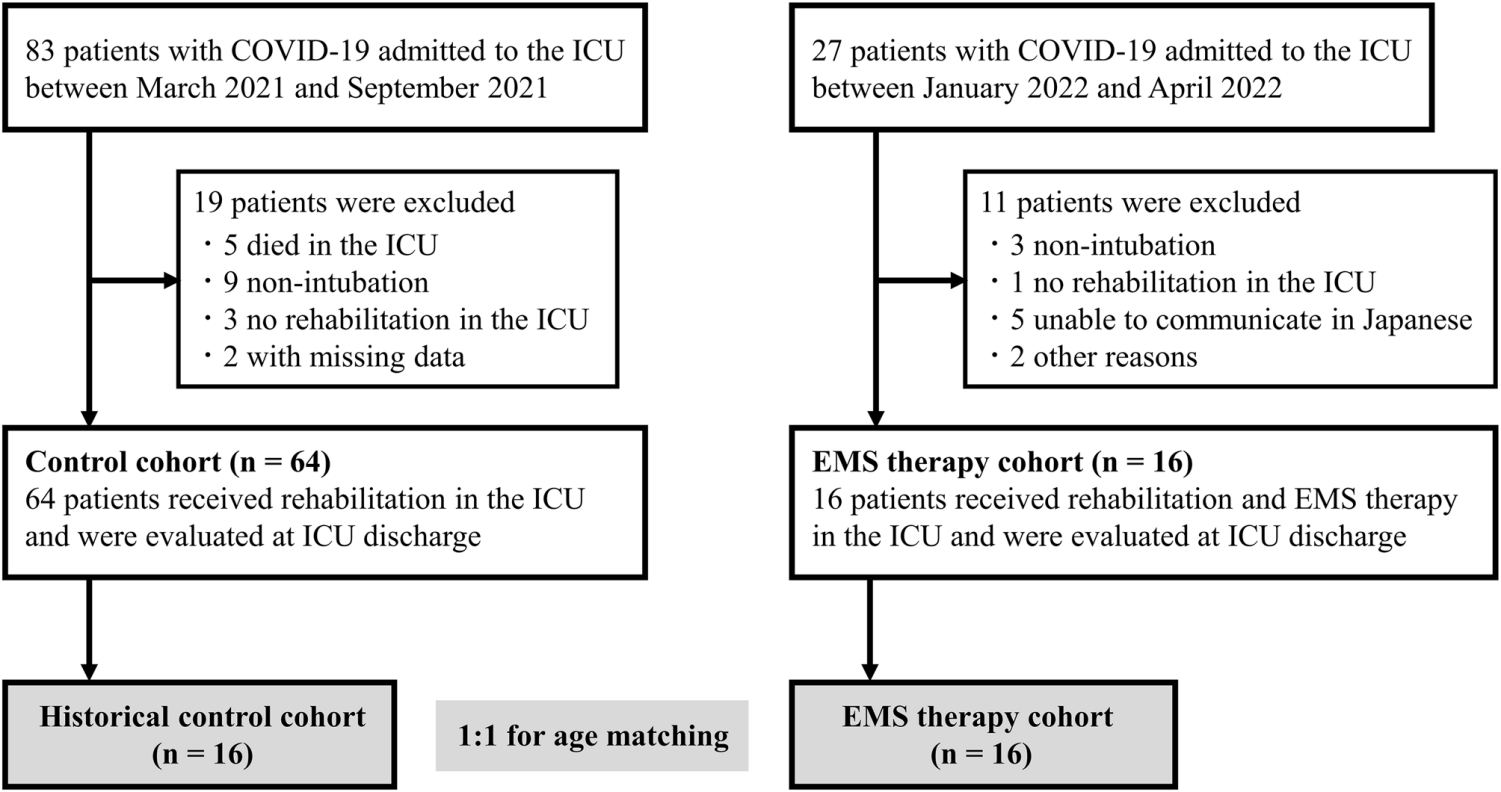
Flow diagram for inclusion of patients in the study. COVID-19, coronavirus disease 2019; EMS, electrical muscle stimulation; ICU, intensive care unit.

During the study period from March 2021 to April 2022, 110 consecutive critically ill patients with laboratory-confirmed COVID-19 were admitted to the ICU of Nagoya University Hospital. The final analysis was performed using data from 16 patients in the EMS therapy group and 16 age-matched historical controls with a median age of 71 years (81.2% male) (Fig. 1). There were no significant differences in baseline clinical characteristics between the two groups, except in vaccination status, SOFA score, and APACHE II score (Table 1).

**Table 1:** Baseline characteristics.

Factor	Historical control	EMS therapy	P value
	Cohort (n = 16)	Cohort (n = 16)	
Age (years)	71 [65–73]	72 [65–73]	0.835
≥ 65 (%)	12 (75.0)	12 (75.0)	1.000
Male (%)	14 (87.5)	12 (75.0)	0.654
BMI (kg/m^2^)	23.6 [21.9–25.5]	24.4 [23.6–27.0]	0.522
≥ 25	5 (31.2)	7 (43.8)	0.716
Vaccination status			
Unvaccinated	15 (93.8)	8 (50.0)	0.015
4C mortality score	14 [12–15]	14 [12–15]	0.732
SOFA score	11 [10–12]	10 [8–10]	0.025
APACHE II score	25 [19–26]	19 [18–22]	0.035
PaO_2_/FiO_2_ ratio	81 [65–162]	124 [95–158]	0.665
Clinical frailty scale score	3 [3–3]	3 [3–6]	0.185
Transfer from other hospitals (%)	13 (81.2)	11 (68.8)	0.685
Charlson comorbidity index	2 [0–4]	2 [1–2]	0.833
ICU therapy (%)			
Sedative drug			
Propofol	13 (81.2)	15 (93.8)	0.600
Midazolam	4 (25.0)	5 (31.2)	1.000
Dexmedetomidine	16 (100.0)	16 (100.0)	1.000
Use of norepinephrine	16 (100.0)	15 (93.8)	1.000
Inotropic treatment	8 (50.0)	7 (43.8)	1.000
ECMO	2 (12.5)	1 (6.2)	1.000
Prone positioning	6 (37.5)	8 (50.0)	0.722
Tracheostomy	4 (25.0)	6 (37.5)	0.704
Steroid pulse therapy (methylprednisolone 1000 mg/day for 3 days)	9 (56.2)	14 (87.5)	0.113
Neuromuscular blockade	4 (25.0)	8 (50.0)	0.273
Cumulative dose of rocuronium (mg)	0 [0–248]	63 [0–826]	0.251

Values are expressed as n (%) or median [interquartile range].

APACHE II, Acute Physiology and Chronic Health Evaluation II; BMI, body mass index; ECMO, extracorporeal membrane oxygenation; ICU, intensive care unit; PaO_2_/FiO_2_, partial pressure of oxygen/fraction of inspired oxygen; SOFA, sequential organ failure assessment; 4C, Coronavirus Clinical Characterisation Consortium.

**Figure 2:**
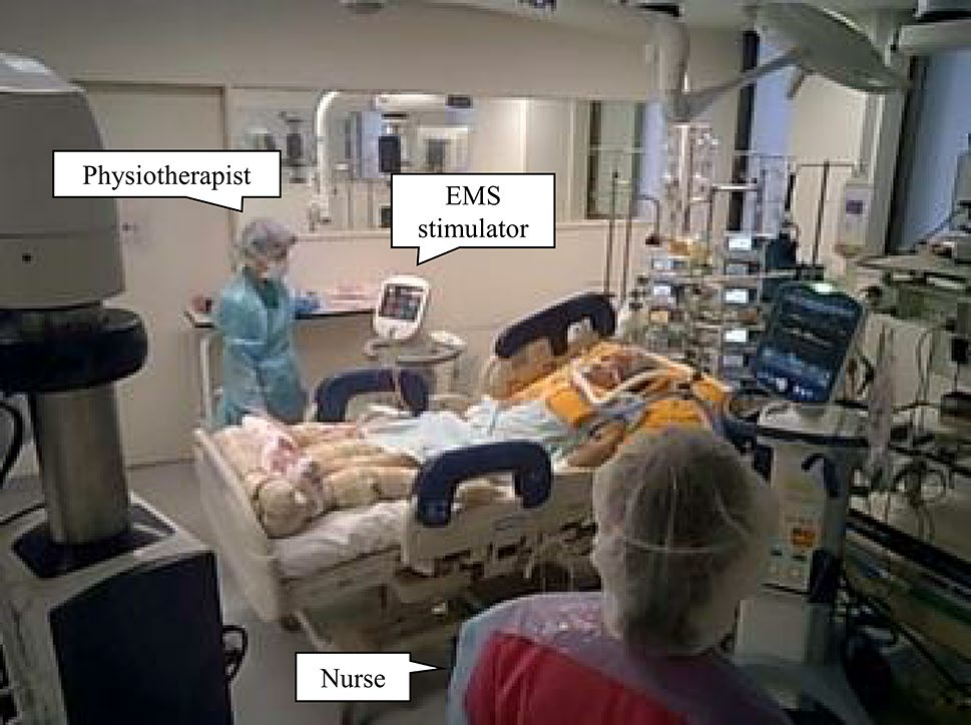
Electrical muscle stimulation for patients with severe COVID-19. COVID-19, coronavirus disease 2019; EMS, electrical muscle stimulation. This figure was provided after informed consent and permission were received from the patient.

In the EMS therapy group, EMS therapy was initiated 3.2 ± 1.4 days after ICU admission, and patients completed a mean of 6.2 ± 3.7 EMS sessions (median, 5 sessions; total, 99 sessions) (Fig. 2). Five patients completed the 2-weeks of EMS intervention before ICU discharge. Two patients dropped out because they complained of muscle discomfort induced by EMS. Thus, the completion rate of the planned sessions until ICU discharge was 87.5%. EMS was applied to the biceps brachii, quadriceps femoris, and the gastrocnemius muscles at intensities of 30 ± 10 milliampere peak (mAp), 51 ± 9 mAp, and 37 ± 9 mAp, respectively. Patients with severe COVID-19, including those on extracorporeal membrane oxygenation (ECMO) support or placement in the prone position, received EMS therapy (Fig. S1). No alterations in vital signs (heart/respiratory rate, blood pressure, and blood oxygen saturation) or adverse events occurred during EMS. There were no cases of hospital-acquired SARS-CoV-2 infection among the medical staff during the study period.

There was no significant difference in median MRC sum score at discharge from the ICU between the EMS therapy group and historical controls (51 points [IQR 42–55] vs. 53 points [IQR 46–59], respectively; P = 0.439). Physical function at ICU discharge, including rates of MRC sum score < 48 points (31% vs. 25%, respectively; P = 0.680) and handgrip strength (7.3 kg [IQR 4.2–15.1] vs. 11.6 kg [IQR 8.4–15.9], respectively; P = 0.239), showed no significant differences between the two groups (Table 2). There were no significant differences in clinical outcomes, including number of days taken to first mobilization, number of ventilator-free days, length of stay in the ICU, ICU mobility scale at ICU discharge, and rate of discharge home between the two groups (Table 2).

**Table 2:** ICU outcomes and physical function at ICU discharge.

Factor	Historical control	EMS therapy	P value
	cohort (n = 16)	cohort (n = 16)	
Time to first mobilize (day)	9 [7–11]	8 [5–9]	0.147
ICU LoS (day)	11 [10–16]	12 [9–26]	0.820
Duration of sedation (day)	8 [5–11]	6 [4–9]	0.691
Duration of ventilation (day)	9 [6–14]	8 [5–9]	0.372
Physical function at ICU discharge			
MRC sum score (point)	53 [46–59]	51 [42–55]	0.439
< 48 points	4 (25.0)	5 (31.3)	0.680
Handgrip strength (kg)	11.6 [8.4–15.9]	7.3 [4.2–15.1]	0.239
Grip and release test score (/10 s)	14 [9–24]	16 [10–25]	0.580
Foot Tapping test score (/10 s)	13 [9–16]	15 [8–23]	0.533
ICU Mobility Scale at ICU discharge	3 [3–4]	3 [3–4]	0.760
Post-ICU LoS (day)	6 [4–16]	4 [0–18]	0.378
Readmitted to ICU (%)	2 (12.5)	2 (12.5)	1.000
Discharged to home (%)	6 (37.5)	5 (31.3)	1.000

Values are expressed as n (%) or median [interquartile range].

ICU, intensive care unit; LoS, length of stay; MRC, Medical Research Council.

## Discussion

This study showed that EMS therapy of the muscles of the upper and lower extremities added to early rehabilitation compared with early rehabilitation alone in patients admitted to the ICU due to severe COVID-19 with respiratory failure, and did not result in improved global muscle strength as assessed by the MRC score at ICU discharge, and was not associated with any adverse events. There were also no significant differences in important clinical outcomes, such as the number of ventilator-free days and ICU mobility scale at ICU discharge, between the EMS therapy group and age-matched historical controls.

Consistent with previous studies in critically ill patients, EMS was initiated a mean of 3.2 ± 1.4 days after ICU admission for COVID-19 patients with IMV, ECMO, and/or placement in the prone position, and was accompanied by neither effects on vital signs nor adverse events in the present study^13^, suggesting that acute-phase intensive EMS therapy is safe for use in critically ill COVID-19 patients admitted to the ICU. However, our findings were inconsistent with a previous meta-analysis indicating that EMS reduces ICU-acquired weakness and increases muscle strength during ICU admission^13^. As these previous studies did not discuss administration of EMS to patients with COVID-19, it was not possible to perform direct comparisons of the effects of EMS with the present study.

There have been few studies of the effects of EMS therapy in patients with COVID-19. In a previous RCT, application of EMS to the gastrocnemius muscles for up to 14 days was accompanied only by improvements in lower extremity muscle condition, e.g., ankle muscle strength and endurance, in critically ill patients with COVID-19 admitted to the ICU^30^. In another study, application of EMS to the quadriceps femoris muscles for 7 consecutive days only increased muscle strength assessed according to the MRC score and function in patients with severe COVID-19 during ICU admission, although they did not include a control group for comparison^31^. The results of the present study indicated that the application of EMS to the biceps brachii, quadriceps femoris, and gastrocnemius muscles for up to 2 weeks (median 5 days) was not accompanied by a decrease in occurrence of ICU-acquired weakness (i.e., MRC score < 48 points) and improved physical function and mobility at discharge from the ICU in patients with COVID-19 requiring IMV. Early additional muscle exercise may not improve muscle function in the most fragile patients with severe inflammation-induced muscle protein breakdown^25^. As a previous RCT suggested that the application of EMS for 7 days was required to prevent muscle atrophy and weakness in critically ill patients^32^, the duration of treatment in the present study may not have been sufficient to observe improvements in the outcomes of our patients. The effects of EMS therapy on physical function may have been attenuated by the mobilization program in the present study as we compared the effects of early rehabilitation with addition of EMS to early rehabilitation alone and more than 75% of our patients could sit on the edge of the bed or better before discharge from the ICU. Moreover, as more than 70% of our patients had MRC score ≥ 48 points at discharge from the ICU and the highest possible score is 60 points, this suggests that a ceiling effect^33^ may have prevented detection of differences between groups. Further studies are required to determine the optimal frequency and duration of EMS therapy and the most suitable method for physical assessment to improve clinical outcomes in patients with severe COVID-19 requiring ICU admission.

This study had several limitations, the most important of which was the small sample size, which may have been underpowered for detection of some of the clinical characteristics and outcomes. In addition, this was not a RCT but compared data from patients before and after the introduction of EMS therapy in our hospital. Because of several advances in the treatment of critically ill patients that affect the type of treatment used, it may be risky to compare data with historical controls. Further RCTs are required to determine the effects of EMS. In addition, the optimal EMS configurations and parameters for patients with severe COVID-19 remain to be determined. As we measured physical function only at discharge from the ICU, the effects of EMS may have been influenced by physical function before admission. Therefore, it was considered necessary to perform an initial assessment upon awakening to observe differences between the two time points. We did not assess oedema, blood flow, and basal metabolic rate. In addition, we did not measure muscle mass or examine mental health, which may be important considerations in patients with severe COVID-19. Finally, this was a single-center study in a population of Asian patients, thus limiting the generalizability of our findings to other populations. However, the single-center setting ensured that similar sedation and ventilator weaning protocols were applied in both groups, and so may also be seen as a strength of this study.

## Conclusions

The results of the present study indicated the safety of EMS therapy in critically ill patients with COVID-19 in the ICU setting, but adding EMS of the upper and lower muscles to a standardized early rehabilitation program did not improve either physical function or clinical outcomes at discharge from the ICU in patients with COVID-19 requiring IMV.

### Supplementary Information

Supplementary Figure S1.

## Data Availability

The datasets used and/or analyzed during the current study are available from the corresponding author on reasonable request.

## Abbreviations

COVID-19: Coronavirus disease 2019
SARS-CoV-2: Severe acute respiratory syndrome coronavirus 2
IMV: Invasive mechanical ventilation
ICU: Intensive care unit
EMS: Electrical muscle stimulation
RCT: Randomized controlled trial
APACHE II: Acute Physiology and Chronic Health Evaluation II
SOFA: Sequential Organ Failure Assessment
MRC: Medical Research Council
IQR: Interquartile range
