# Supplementary material for: Effects of acute phase intensive electrical muscle stimulation in COVID-19 patients requiring invasive mechanical ventilation: an observational case-control study
Source: Sci Rep. 2024 Mar 4;14:5254. doi: 10.1038/s41598-024-55969-8 (PMC10912433; doi:10.1038/s41598-024-55969-8)
Supplement: Supplementary file 1 — Supplementary Figure S1. [file 41598_2024_55969_MOESM1_ESM.pdf]

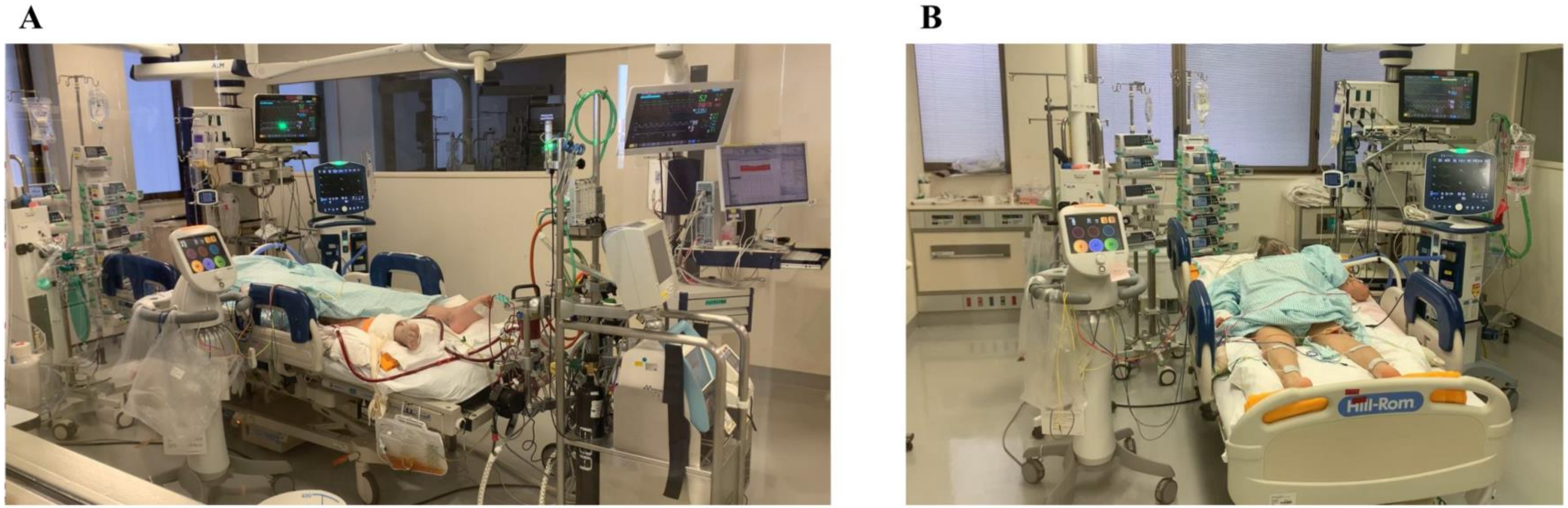

Figure S1. Electrical muscle stimulation for patients with severe COVID-19 (A) on ECMO support and (B) placed in the prone position. COVID-19, coronavirus disease 2019; ECMO, extracorporeal membrane oxygenation. This figure was provided after informed consent and permission were received from the patients.
